# Quantifying mutual hesitation and identifying kinematic predictors in near-collision avoidance in walking

**DOI:** 10.1038/s41598-026-59801-3

**Published:** 2026-07-07

**Authors:** Kazuyuki Sato, Johannes Keck, Rouwen Cañal-Bruland

**Affiliations:** Department for the Psychology of Human Movement and Sport, Friedrich Schiller University Jena, Seidelstraße 20, 07749 Jena, Germany

**Keywords:** Collision avoidance, Mutual anticipation, Kinematics, Social interaction, Cross-recurrence quantification analysis, Neuroscience, Psychology, Psychology

## Abstract

Humans navigate crowded environments by anticipating others’ movements. When such mutual anticipation fails, this may result in collisions or near-collisions. Previous research on mutual anticipation in collision avoidance has highlighted the importance of kinematic information. However, it remains unclear how full-body kinematics support mutual anticipation in truly interactive near-collision avoidance scenarios.　We introduce cross-recurrence quantification analysis (CRQA) as a novel method to quantify the degree of mutual hesitation (i.e., spatiotemporal overlap between walkers) in near-collision avoidance. Additionally, we examined which pre-avoidance, inter-participant kinematic relationships predict the degree of mutual hesitation. Forty-five dyads (i.e., 90 participants) walked toward each other and aimed at avoiding collision in two differently sized avoidance zones (20 cm, 80 cm). Results revealed that the mean Recurrence Rate in mutual hesitation trials was 37.97 (± 23.2) and 41.95 (± 25.5) in the respective collision avoidance zones. Statistical Parametric Mapping showed that inter-participant head-angle differences diverged earlier than the shoulder or pelvis in non-mutual hesitation trials. Smaller head-angle differences 0.3–0.2s (20 cm) and 0.2–0.1s (80 cm) before avoidance onset predicted a higher degree of mutual hesitation. We conclude that failures in inferring avoidance direction from head kinematics may be linked to mutual anticipation errors that result in mutual hesitation.

**Supplementary Information:**

The online version contains supplementary material available at 10.1038/s41598-026-59801-3.

## Introduction

Navigating public environments and avoiding collisions with cars, bicycles and pedestrians are fundamental to our daily lives. Interestingly, even in an extremely complex environment such as the Shibuya Crossing in Tokyo, pedestrians fluidly navigate shared open spaces while successfully avoiding collisions most of the time. Although actual collisions are rare, pedestrians sometimes encounter near-collisions in which mutual anticipation fails—pedestrians incorrectly anticipate each other’s intended passing side—leading to so-called mutual hesitations^1^. Such mutual hesitations reflect moments when the risk of collision is likely to increase. Understanding why mutual hesitation occurs is practically important because such hesitation reflects a breakdown of smooth mutual anticipation during collision avoidance and may become particularly relevant in spatially constrained pedestrian interactions^1,2^. This knowledge is also increasingly relevant for technology. For instance, as robots or VR avatars become more common in everyday environments, we need to understand which behavioral cues and decision rules enable smooth, predictable, and human-compatible navigation. Therefore, identifying the mechanisms underlying mutual hesitation is crucial from both a theoretical and practical perspective.

Previous research has convincingly demonstrated that mutual anticipation is key for smoothly avoiding collisions^1,3^. In this process, body kinematics are suggested to play a pivotal role. Specifically, in a study of a head-on collision avoidance scenario, Murakami et al. (2022) disrupted mutual anticipation (by operating a phone) or mutual gaze (by wearing sunglasses). They found that coordination was degraded only in the former, but not the latter condition, indicating that mutual anticipation may rely on bodily (i.e., kinematic) cues. This finding is consistent with evidence that action intentions can be inferred from body motion^4–8^. Therefore, examining the kinematics of pedestrians in collision avoidance situations is crucial for uncovering the mechanisms underlying mutual anticipation.

Research examining kinematic cues that may be critical for mutual anticipation during collision avoidance points to several candidates. To start with, in avoidance tasks with a VR avatar, the orientation of the agent’s head has been shown to strongly guide participants’ avoidance choices^9^. Gaze direction similarly biases this avoidance strategy, with observers tending to pass on the side opposite to the other’s gaze^10^. This may be a reasonable strategy given the tendency for the gaze and head to be directed first to the intended travel direction^11,12^. Another study suggested that waist rotation provides an early cue to avoidance direction, because waist rotation preceded mediolateral displacement during avoidance^13^. Leg movements—particularly step timing and direction—have also been proposed as important cues for inferring avoidance direction^14^. Given that changes in shoulder orientation precede changes in walking direction (velocity direction) by approximately 100 ms in collision-avoidance situations^15^, shoulder orientation also seems a plausible candidate as an anticipatory cue.

Despite the fact that the above-mentioned research has significantly advanced our knowledge regarding the importance of mutual anticipation in collision avoidance and the particular role of kinematic information therein, important gaps remain to be addressed. First, to the best of our knowledge, thus far, no studies have examined which kinematic cues support mutual anticipation from a full-body measurement perspective. Given that changes in walking direction typically emerge first in gaze, followed by the head and the trunk, it is essential to analyze whole-body kinematics and their temporal unfolding. In other words, which kinematic signals are associated with or predictive of mutual hesitation are not sufficiently understood yet. Second, most previous studies have not implemented truly interactive situations. However, to gain a better understanding of mutual anticipation, applying paradigms that use dynamic, real-time interactions is paramount^16^. Having said this, some researchers have previously used pre-programmed, non-responsive avatars or animations. However, these avatars lacked the capacity for genuine mutual adaptation. Other researches, such as Willems et al. (2020) and Murakami et al. (2022), have investigated behavior in highly natural settings requiring mutual anticipation; yet, these studies did not capture full-body kinematics. In addition, a method to quantify mutual hesitation at the dyadic level also remains to be developed yet. To our knowledge, the only exception is a study by Honma et al. (2015) that was aimed at experimentally examining mutual hesitation and its underlying mechanisms. The authors analyzed approaching walkers’ trajectories. However, they defined mutual hesitation by summing or averaging individual measures such as hesitation times and avoidance-direction reversals in the walking trajectories (i.e., initially moving to one side and then switching to the other), without capturing bidirectional (i.e., mutual spatiotemporal) coupling within the dyad. To address this latter gap in the literature, we propose a novel approach to quantify mutual hesitation.

More specifically, the aims of this study were twofold: (i) to establish a parsimonious method for defining and quantifying mutual hesitation in head-on collision avoidance by means of cross-recurrence quantification analysis (CRQA), which has increasingly been exploited in other domains of human movement and behavior analysis recently^17–20^; (ii) to examine the mechanisms underlying mutual hesitation from a whole-body kinematics perspective in an interactive collision avoidance scenario by identifying the pre-avoidance kinematic features that allow for predicting the degree of mutual hesitation.

We propose CRQA as a method to detect and quantify mutual hesitation in a near-collision scenario because it provides an elegant way to quantify the temporal and spatial synchronization between two time series data^21–25^. After quantifying and classifying mutual hesitation or non-mutual hesitation, we then utilized statistical parametric mapping (SPM) analysis, which has increasingly been adopted in human movement research^26–30^. SPM analysis allowed us to identify which kinematic features are most closely associated with the earliest stage of mutual hesitation. Finally, we used linear mixed-effects models (LMMs) to test which kinematic variables predict the degree of mutual hesitation, and at what time points these predictive relationships emerge.

## Methods

### Participants

Forty-five dyads (90 participants; mean age = 22.3 years, SD = 3.6) participated in the present study. We balanced out any combinations of gender as we strived for generalizable findings (i.e. across gender) regarding mutual anticipation. Accordingly, and similar to previous studies such as^1^, 15 dyads were female only, 15 dyads were male only, and 15 dyads were mixed gender. No a priori power analysis was conducted regarding the sample size. Because mutual hesitation occurs spontaneously and relatively infrequently, the effective number of observations depended on the number of hesitation events obtained rather than only on the number of dyads. Participants were recruited via e-mail, flyers, and in person on the university campus. Participants were all residents of a right-side traffic region (Germany). This study was carried out in accordance with the standards of the Declaration of Helsinki. All participants received a full explanation of the study and provided written informed consent. The ethical Commission of the Faculty of Social and Behavioural Sciences at Friedrich Schiller University Jena approved the study protocol (approval no. FSV 24/093).

### Experimental setup

**Fig. 1:**
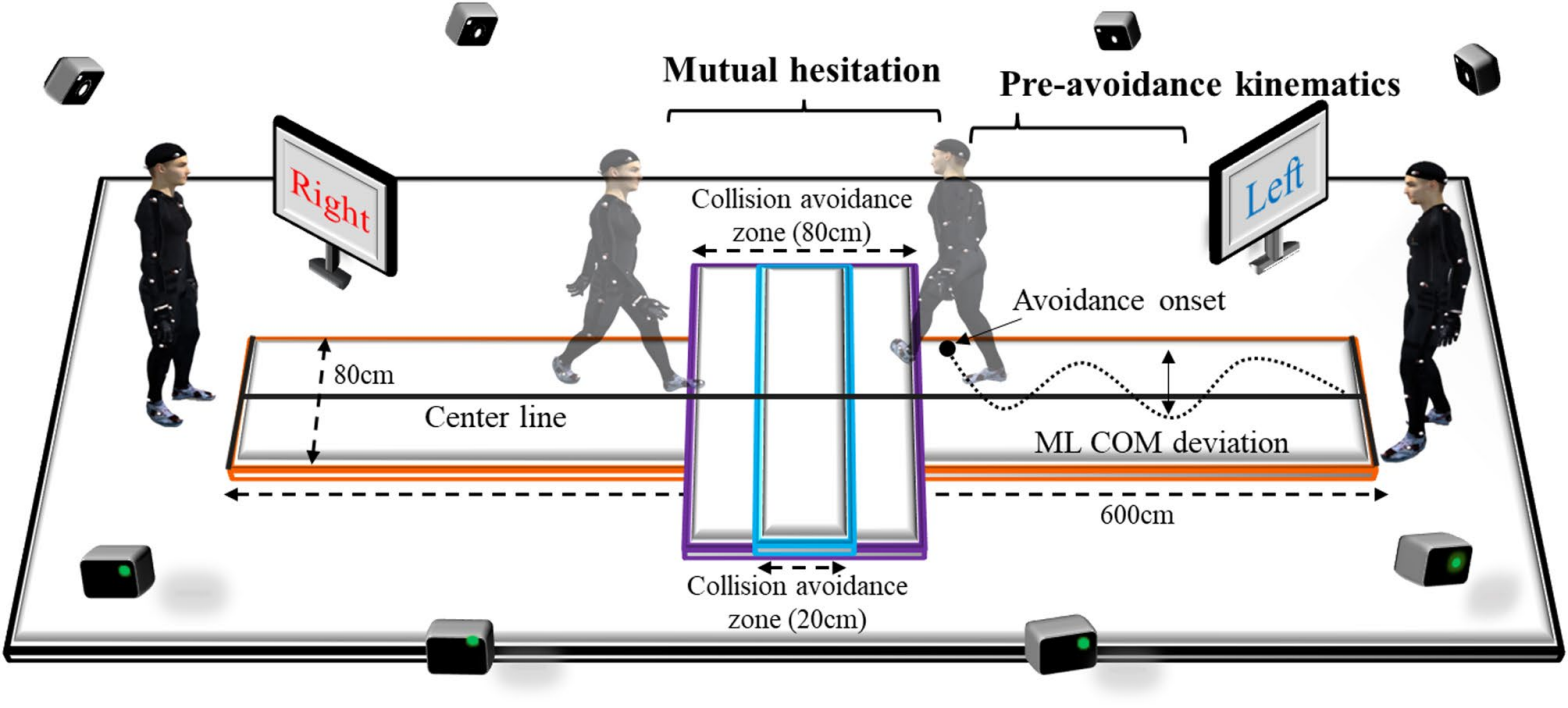
The experimental set-up. Masking tape was used to create a corridor-like walking space with a length of 6 m and a width of 80 cm. A center line was drawn vertically in the middle of the walking space. Two different distance areas, where the participants could move freely, were set in the middle of the walking space: (1) 20 cm length indicated by the light blue lines, and (2) 80 cm length indicated by the purple lines. The width of the collision avoidance zone was 170 cm. In an additional experimental condition (i.e., conducted after the main experiment, see Procedure), two displays were used to instruct participants.

The experiment setup closely resembled the design of Honma et al. (2015) (see Fig. 1). In fact, to additionally manipulate the time pressure, we implemented two differently sized collision avoidance zones (20 cm and 80 cm). Based on previous evidence that longer pre-event distance can increase opportunities for mutual prediction and hesitation, whereas greater decision time can sometimes promote delayed decisions or paralysis-by-analysis^1,31^, we expected that the longer distance condition would allow more mutual anticipation and thereby modulate mutual hesitation, whereas the shorter distance condition would impose stronger time pressure and promote more reactive avoidance. The distances were determined based on the study by Honma et al. (2015) and our own pilot experiments. They used 20 cm and 200 cm collision avoidance zones and noted the absence of intermediate distances as a limitation. In our pilot study, we conducted the same experimental task as the main task in the present study while varying only the length of the collision avoidance zones. The results showed that longer distances (200 cm, 140 cm) allowed participants to resolve collision risk too easily, decreasing the occurrence of mutual hesitation. We therefore only included 20 cm (short) and 80 cm (medium) as the experimental distances. Whole-body motion was recorded using a 16-camera OptiTrack motion-capture system (NaturalPoint, Inc., Corvallis, USA) at 120 Hz. Participants wore a motion-capture suit with 57 reflective markers; the markers used for the present analyses included pelvis markers for COM estimation, head markers for head orientation, shoulder/chest markers for shoulder orientation, and anterior/posterior pelvis markers for pelvis orientation.

### Procedure

In the collision avoidance task, participants were instructed to walk straight toward the collision avoidance zone at their preferred walking speed while keeping their center of mass (COM) on the center line (see Fig. 1). Each trial began with a start signal and a countdown from the PC saying ‘3, 2, 1, start’. Once the signal was given, participants started to walk. When entering the collision avoidance zone, participants were required to step onto the designated entry line corresponding to their assigned distance conditions. At this moment, the participants were still separated by more than the nominal length of the collision avoidance zone because only one foot had reached the entry line. In the main experiment, they were free to choose their avoidance direction within the collision avoidance zone, and no specific instructions were given regarding their behavior or the timing of avoidance initiation or deviation onset. After exiting the collision avoidance zone, participants proceeded to the goal line while keeping their COM on the center line. Upon reaching the goal line, the participants waited for the start of the next trial. For each distance, participants completed 20 trials, resulting in 40 trials in total. Considering the complexity of the task, the 80 cm condition was conducted first, allowing participants to gradually become familiar with the less complex distance to the more complex distance conditions.

After completing the main experiment, participants performed an additional experimental condition in which explicit instructions regarding the avoidance directions were provided so as to cause a large number of near-collisions. This condition was necessary to generate sufficient near-collision data for estimating and calibrating the internal CRQA parameters. In general, such parameters are chosen in a data-driven way, but mutual hesitation occurred too rarely in the main experiment, making it challenging to apply the traditional data-driven approach. Thus, in this second condition, after stepping on the entry line, participants had to follow the instruction of avoidance direction indicated on the display (e.g., right or left). By providing the instructions, we experimentally induced trials in which the two participants were set onto the same avoidance directions (overlap trials) and trials in which they chose opposite directions (non-overlap trials). Participants again completed both distance conditions (20 cm and 80 cm). For each distance condition, they performed 40 trials with a 50% probability of overlapping avoidance direction (20 overlap trials and 20 non-overlap trials), resulting in 80 trials per dyad. The order of overlapping and non-overlapping trials was randomly assigned. During the experiment, participants were free to take breaks at their preferred times. Prior to the main experiment, a practice session was conducted to ensure that participants fully understood the task. After providing a scripted explanation, each condition was practiced until the participants demonstrated a clear understanding of the task requirements.

### Data analysis

#### Data preprocessing

We excluded one dyad’s data because one participant’s pelvis marker detached during the trials, resulting in substantial missing pelvis data that required extensive interpolation and rendered the kinematic estimates unreliable. To reduce high-frequency noise, the raw data were low-pass filtered using a fourth-order Butterworth filter (cutoff frequency = 3 Hz, normalized frequency = 0.05 at 120 Hz sampling) beforehand. Zero-phase filtering (forward–backward) was employed to avoid phase distortion. This process was performed using MATLAB version R2024a (The MathWorks Inc., Natick, MA, USA).

#### Phase definitions: observation and reaction phases

The previous studies^32,33^ classified collision-avoidance behavior into an observation, reaction, and regulation phase. Following this framework, we adopted a data-driven approach in which all phase boundaries (thresholds) were determined by the trajectories, computed from four pelvis markers, as detailed below. The observation phase began when participants moved forward by 20 cm. This was to exclude noise during quiet standing before gait initiation. The observation phase ended at the onset of avoidance for either participant (for example, see Fig. 1). Similar to previous studies^34–37^, the avoidance onset was determined from the medial-lateral (ML) deviation of the COM. Specifically, we extracted the maximum ML COM deviation (see Fig. 1) of each participant until entering the collision avoidance zone across trials and set the avoidance-onset threshold to the mean plus two standard deviations (SD). These maximum values had a mean of 10.3 cm and an SD of 6.2 cm, resulting in an avoidance-onset threshold of 22.7 cm. For instance, when either participant deviated from the centerline by 22.7 cm, that moment was regarded as the avoidance onset. To define the start of the reaction phase, we first computed the inter-participant anterior-posterior (AP) clearance at the moment of avoidance onset in each trial. The mean AP clearance at this moment was 74.2 cm for the 20 cm distance condition and 116.8 cm for the 80 cm distance condition. These values were used as thresholds, and the reaction phase was defined to start when the inter-participant AP clearance fell to or below the corresponding threshold. The end of the reaction phase was determined based on the inter-participant ML clearance between participants within the collision avoidance zone. We computed the mean maximum ML clearance across all trials and set the threshold to the mean − 2 SD, yielding 35.0 cm. This threshold means that both participants were sufficiently separated and the collision risk was resolved. We used a common threshold to apply the same endpoint criterion across trials and to restrict the CRQA analysis to the period in which collision risk was still present.

To avoid potential bias, we used these common pooled thresholds rather than participant-specific or trial-specific thresholds. The analyses required one common avoidance-onset time point per trial, and avoidance behavior could be asymmetric, with one participant showing a clear lateral deviation while the other did not. In such cases, participant-specific thresholds could bias onset detection, whereas trial-specific thresholds would be unstable because clear ML COM deviations were not always observed, particularly during mutual hesitation. We therefore adopted a pooled conservative threshold that applied the same criterion across all participants, dyads, and trials. This definition may have detected avoidance onset slightly later than individualized retrospective methods^34^ and may have missed subtle early adjustments. However, because the same criterion was applied across all trials and conditions, the relative comparisons between mutual hesitation and non-mutual hesitation trials remain interpretable.

#### Trajectory analysis

As mentioned before, after the main experiment, we needed to run an additional condition in which participants were instructed about the avoidance direction to increase the likelihood of mutual hesitation. Importantly, this condition was necessary and exclusively used to determine the internal CRQA parameters in a data-driven manner (i.e., as an independent calibration dataset). However, even in trials where the instructions should have induced overlapping avoidance directions, participants sometimes coordinated their movements well, and their trajectories did not overlap. Therefore, we first analyzed their trajectories to precisely classify all trials into overlap and non-overlap trials. The trajectory direction of both participants was calculated from their velocity vectors at each time frame to determine whether they were moving spatially in the same direction at the same moment. The overlap was determined based on the inter-participant heading angle. For instance, when both participants were proceeding in the same direction, those trials were regarded as the overlap trials. To exclude coincidental overlaps during normal walking, we introduced a threshold of heading angle between participants. Specifically, for each participant, we computed the mean and SD of the maximum heading angle within one gait cycle during the observation phase (mean = 11.65°, SD = 4.8°). In research on human gait^38–40^, it is common to define the normal range of a gait parameter, or the boundary between normal and abnormal gait, as mean ± 2 SD, which corresponds to approximately the 95% normal limits under an assumption of normality. Following this, we treated mean + 2 SD as the maximum heading deviation that could occur during normal straight walking, and we interpreted any larger heading deviation than the threshold as an intentional directional change. This procedure yielded a threshold of 21.2° per participant. Then, since there were two participants, the value was doubled to obtain 42.4°. In other words, when the angle between the two walking directions was 137.6° or greater (180° − 42.4° = 137.6°), i.e., nearly parallel, this was regarded as the maximum angle that could occur during normal walking and therefore had to be classified as a non-overlap trial. We then defined overlap as trials in which time frames were observed in which the angle between the two walking directions was less than 137.6°. In addition, time frames in which the risk of collision between the two participants was considered to be resolved (i.e., with an ML clearance of 35.0 cm or greater), and time frames in which no apparent change in walking direction occurred for each participant (11.6° or less), were excluded from the count of the overlap. Based on the results of this trajectory analysis, each trial was labeled either as ‘overlap trial’ or ‘non-overlap trial’.

### Cross-recurrence quantification analysis (CRQA)

In CRQA, two time series of data are reconstructed in a high-dimensional phase space to assess the synchrony between the two data points, and recurrence patterns are quantified, allowing us to define the degree of mutual hesitation from the trajectories of two participants. For example, in our case, we applied CRQA to the ML COM of the two participants and quantified the degree of synchronization between their lateral avoidance movements in time and space. To run CRQA, three internal parameters need to be determined in a data-driven manner: delay (τ), embedding dimension (*m*), and radius (ε). The delay is the time lag used for reconstructing time series data into a high-dimensional space. In the process of CRQA, it constructs the high-dimensional states by aligning ‘current state, slightly past state, and further past state’ from both time series data. Selecting the appropriate lag captures an accurate estimate of the recurrence pattern. When the lag is too small, the redundancy in the dynamics’ continuity increases, leading to an overestimation of synchrony. By contrast, when the lag is too wide, the dynamics between the preceding state and following state become ambiguous, leading to the underestimation of the recurrence. The embedding dimension is the parameter that determines how many time-series states, separated by delays, are treated as a single state. That is, by constructing an *m*-dimensional state vector that concatenates the current and past values separated by a time delay, it is possible to capture not only the instantaneous magnitude but also the short-term temporal pattern (e.g., whether the system is accelerating or decelerating), which is difficult to capture by using instantaneous values alone. If the embedding dimension is chosen too small, distinct states cannot be distinguished precisely, leading to the loss of context and an increase in false detection of recurrence states. In contrast, if the embedding dimension is too big, an unnecessary dimension is increased, leading to amplified noise. The radius (ε) is the neighborhood threshold in phase space to determine recurrences. When the radius is too small, the thresholds become overly strict, yielding too few recurrence points and a fragmented structure (low synchrony). Conversely, setting the radius too large imposes the overly permissive threshold, inducing an excessive number of recurrence points, and inflating estimates of synchrony.

#### Analysis of delay and embedding dimension

The delay and embedding dimensions were determined following the procedures described in previous studies^41,42^. In this parameter selection process, CRQA was applied only to trials that had been classified as overlap in the trajectory analysis, and it was restricted to the reaction phase. The delay was selected as the first local minimum of the average mutual information (AMI). AMI quantifies the statistical dependence between the current value of a signal and a time-delayed version of the same signal. In simple terms, it indicates how well the delayed sample can be predicted from the present sample. The median value between participants was used as a representative value for each distance condition. The embedding dimension was estimated by calculating the most frequent value of the false nearest neighbors rate (FNN). The FNN analysis detects points that appear to be neighbors in a low-dimensional reconstruction but become distant when the embedding dimension is increased. The optimal embedding dimension is reached when the proportion of such false neighbors becomes sufficiently small and no longer changes appreciably with further increases in dimension. The median value was used as a representative value for each distance condition as well. As a result, the optimal delay and embedding dimension were determined to be τ = 3 and m = 2 for the 20 cm distance condition, and τ = 4 and m = 2 for the 80 cm distance condition (for an illustration of the AMI and FNN results, see Supplementary Material, Fig. S1). In subsequent CRQA analyses, these fixed values were used.

#### Analysis of radius

**Fig. 2:**
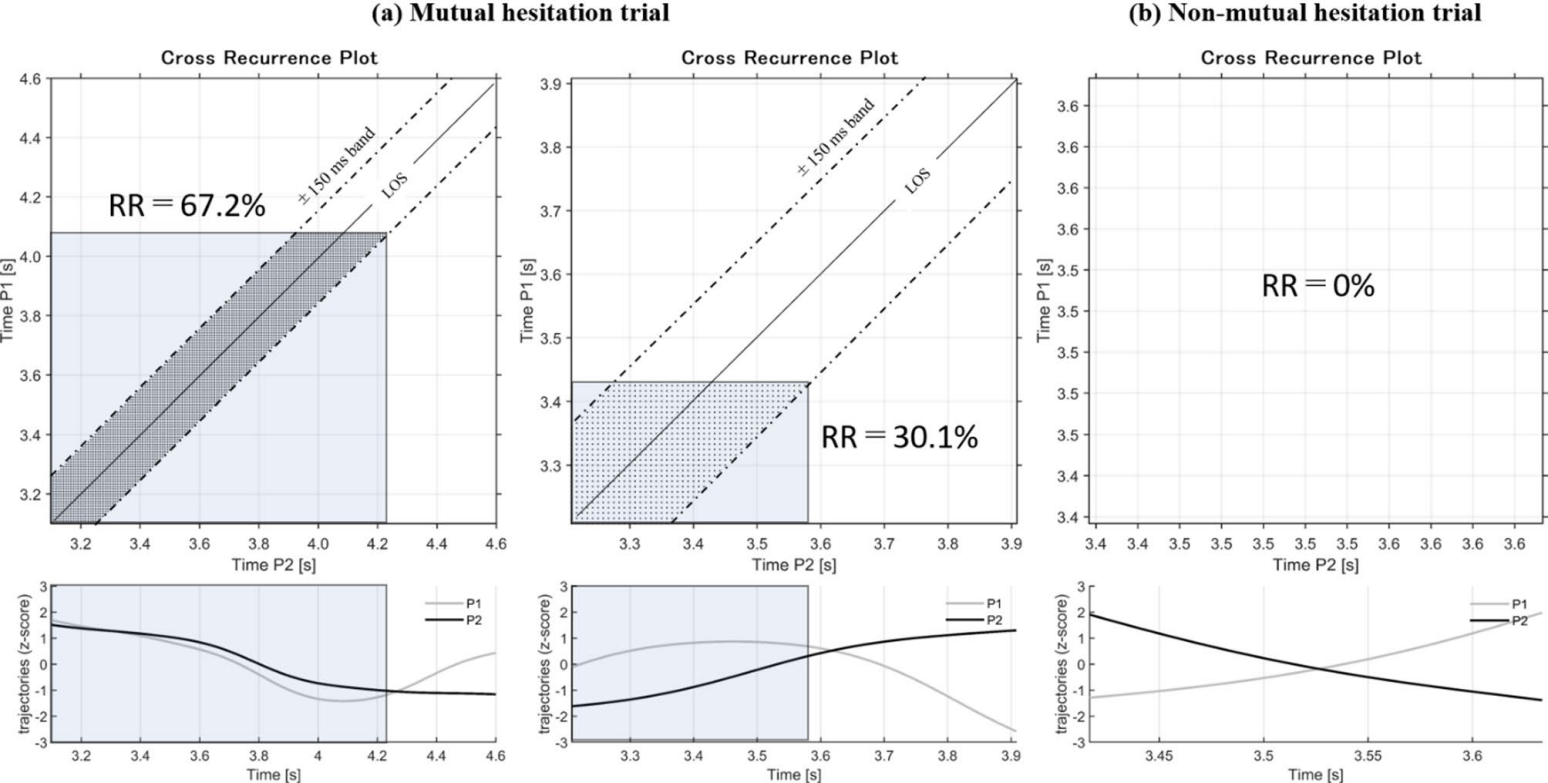
Cross-recurrence quantification analysis (CRQA) of the dyad’s lateral center-of-mass (COM) during the reaction phase. Top: cross-recurrence plot (CRP) with P1 time on the y-axis and P2 time on the x-axis; dots mark recurrent states. The solid diagonal line indicates the line of synchrony (LOS), and the dashed lines indicate the selected near-diagonal bandwidth of ± 150 ms. Bottom: the trajectories of both participants. Each small dot marks a recurrence point (RP), and the recurrence rate (RR) denotes the proportion of recurrence points within the plotted area.

Regarding the radius, we could not follow a common data-driven approach^43^: the radius was adjusted so that the recurrence rate (RR)—the degree of mutual hesitation and the main CRQA measure of synchrony between the two time series—fell within 1–5% (either per trial or with a fixed ε across trials). Because mutual hesitation was rare in the main experiment and the RR was 0 in most trials, standard procedures such as adjusting the RR to 1–5% per trial or averaging the radius across all trials to 1–5% are not reasonable. Furthermore, due to the short time window (the reaction phase in the present study) for applying CRQA, even a slight mutual hesitation would inevitably result in high RR. Therefore, we performed a simple radius sweep to determine the optimal radius. More specifically, we prepared 50 candidate radius values, ran CRQAs with each radius value on trajectories within the reaction phase, and recorded the corresponding RR, classifying each trial as either ‘mutual hesitation’ or ‘non-mutual hesitation’. The present study defined trials where RR > 0 as mutual hesitation and trials where RR = 0 as non-mutual hesitation. To illustrate, representative cross-recurrence plots are shown in Fig. 2. RR is the proportion of recurrence points (RPs) within the plot. Each dot represents an RP, indicating a moment when the two embedded states fall within the radius. Dense populations of dots close to the main diagonal indicate near-synchronous spatiotemporal overlap between the two participants, which was used to identify mutual hesitation. The offset from the diagonal encodes the lead–lag relationship. Specifically, we counted RPs that (i) lay within the main diagonal ± the Theiler window which defines a restricted area where recurrence is evaluated, (ii) corresponded to frames in which the two participants’ lateral movement directions matched, and (iii) occurred when the ML clearance was below the threshold (excluding frames where the collision risk had clearly disappeared). In CRQA, the Theiler window is used to exclude recurrence points along and near the main diagonal (line of synchrony; LOS), because they are often dominated by auto-correlation. However, in our case, recurrence close to the LOS is precisely what we should capture, as it reflects near-simultaneous adjustments of both participants, which we interpret as mutual hesitation. Then, inspired by the concept of diagonal cross-recurrence profile (DCRP)^42–44^, which summarizes recurrence along diagonals parallel to the LOS as a function of temporal lag, we restricted our recurrence quantification to a narrow lag window around the LOS (i.e., the set of near-diagonal lags that is typically excluded via a Theiler window), thereby focusing on near-synchronous coordination. The width of this band (18 samples, corresponding to a lag of up to 150 ms) was chosen based on typical human reaction times for detecting and adjusting locomotor behavior^45,46^. Trials were excluded when the reaction phase was too short to meet the minimum embedded-series length required for CRQA, making recurrence quantification infeasible.

Near-diagonal RPs were counted as mutual hesitation when they reflected near-synchrony of lateral COM overlap before collision risk was resolved. Thus, dense RP patterns close to the diagonal indicate near-synchronous spatiotemporal overlap between P1 and P2. In the left panel (a), synchrony is evident from approximately 3.2 to 4.2 s.

#### Selecting the Optimal Radius Using MCC

We then compared the trajectory-based operational reference classification (overlap vs. non-overlap) with the classifications obtained from the CRQA run with 50 candidate radii as the predictor (mutual hesitation vs. non-mutual hesitation). This trajectory-based classification was not intended as a physiological gold standard, but as an empirical reference for calibrating the radius in the instructed calibration dataset. We calculated the Matthews correlation coefficient (MCC)^47^, which provides a balanced, correlation-based summary robust to class imbalance^48–50^, and selected the radius that yielded the maximum MCC as the optimal value to apply to the main experiment. The calculation formula of MCC is as follows:

$$\:MCC=\frac{TP\cdot\:TN-FP\cdot\:FN\:}{\sqrt{(TP+FP)(TP+FN)(TN+FP)(TN+FN)}}\:$$

When multiple radius values yielded identical MCC, we adopted the smaller radius as a conservative choice, in line with methodological recommendations to avoid overly large recurrence thresholds^51^. As a result, the optimal radius was 4.3 for the 20 cm condition and 2.1 for the 80 cm condition. The corresponding MCC values were 0.97 for the 20 cm condition and 0.95 for the 80 cm condition. The complete set of results on MCC is provided in the Supplementary Material, Table 1.

#### Applying CRQA to main experimental data

After determining the CRQA parameters, we applied CRQA to the main experimental data. RR was used as an index of mutual hesitation, and trials were classified as mutual hesitation (RR > 0) or non-mutual hesitation (RR = 0). We used the same CRQA procedures as above, including RR computation and the same time window.

### Analysis of kinematic predictors

#### Pre-avoidance inter-participant kinematics

**Fig. 3:**
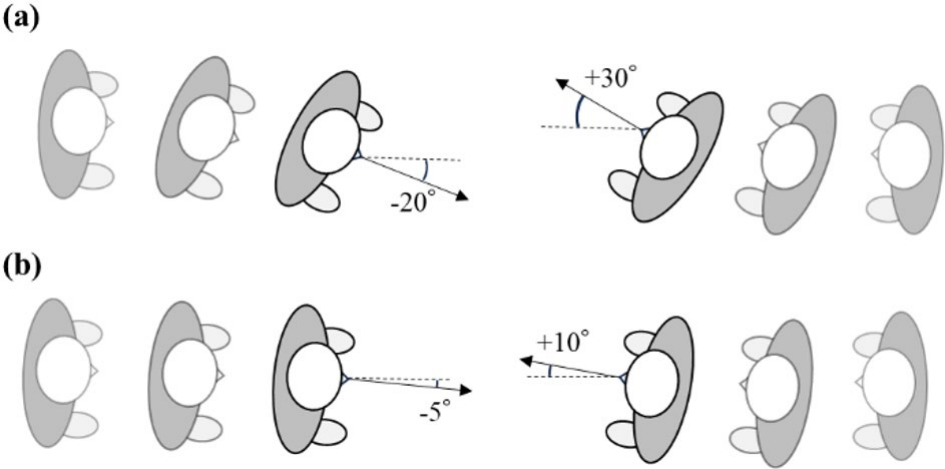
Angular difference between participants’ orientations. The angular difference between the two orientation angles was computed as θ₁ − θ₂. A value of 0° indicates that the participants are perfectly face-to-face, whereas 180° indicates that they are oriented in exactly opposite directions. Examples: (**a**) 30 − (− 20) = 50°, (**b**) 10 − (− 5) = 15°.

To quantify the orientation of each participant’s body, we calculated the forward vectors for (i) the head (forehead–vertex), (ii) shoulders (chest marker relative to the midpoint of left and right acromion markers), and (iii) pelvis (midpoint of anterior pelvic markers relative to the midpoint of posterior pelvic markers) in the horizontal (X–Z) plane (Fig. 3). Based on prior evidence that gaze does not substantially contribute to mutual anticipation in pedestrian collision avoidance^52^ and given the mechanical constraints on foot placement in our task, we focused on these three primary orientations. For each body part vector, we calculated the yaw angle (orientation in the horizontal plane relative to the anteroposterior axis) within a time window of one second prior to the avoidance onset. For every time point, we then calculated the inter-participant angular difference for the head, shoulders, and pelvis. For instance, when both participants were facing in the same direction, the angle was 0°, whereas 180° indicates they faced in exactly opposite directions. To reduce the influence of extreme trials, outliers were identified on the basis of z-scores, and trials with |z| > 3 (exceeding ± 3 SD from the pooled mean across distance conditions) were excluded from subsequent analyses.

#### Covariates

To focus on the effect of kinematics and control the effect of environmental factors, we evaluated the differences in entry timing, ML clearance, and synchronization of the stepping foot, which were examined in the study by Honma et al. (2015). Entry timing was defined as the time at which both participants entered the collision avoidance zone. Large differences in entry timing can create a clear leader–follower relationship. ML clearance was the inter-participant lateral distance of the COM at the moment of entering the collision avoidance zone. Although participants were instructed to walk along the center line, the COM could already be shifted laterally at entry. Synchronization of the stepping foot captured the laterality (left/right) of the foot that first crossed the entry line. We classified trials as ipsilateral when the two participants entered with mirror-opposite feet (i.e., one with the right foot and the other with the left), which implies both were stepping with the foot on the same side of the dyad. In such cases, because people biomechanically tend to initiate a lateral step, we expected a greater likelihood of same-side avoidance. These variables were extracted at the moment when the toe marker of either stepping foot crossed the entry line of the collision avoidance zone. Because the toe markers were attached not exactly at the tip of the toe but around the metatarsal region, a 10 cm margin was applied in the calculations. Unlike Honma et al. (2015), who controlled participants’ walking rhythm, we did not impose constraints on participants’ natural walking rhythm or speed. Therefore, to account for possible effects of walking speed, we additionally calculated two dyadic speed variables: the mean walking speed of the two participants and the absolute inter-participant walking-speed difference during the pre-avoidance window.

### Statistical analysis

#### Outlier removal and data exclusion

We excluded fourteen trials in the 20 cm condition and twenty trials in the 80 cm condition in which CRQA failed to produce valid results because the analysis segment was too short for CRQA, yielding non-finite values for the primary outputs. We also excluded seventeen trials in the 20 cm condition and fifteen trials in the 80 cm condition that were identified as outliers (defined as values above the mean + 3 SD) in the inter-participant angular difference, as well as one trial in the 80 cm condition with missing head-marker data. A detailed breakdown of trial exclusions and the number of trials included in the sensitivity analysis is provided in Supplementary Table S4. To assess whether the exclusion of angular-difference outlier trials influenced the main findings, we additionally conducted a sensitivity check in which these trials were retained but extreme angular-difference values were capped at the predefined quality-control thresholds. The results of this sensitivity check are reported in Supplementary Tables S5 and S6.

#### Pre-avoidance kinematics between mutual hesitation and non-mutual hesitation

To examine the overall inter-participant angular differences between the presence or absence of mutual hesitation, we ran a 2 × 2 ANOVA on each dependent variable (the inter-participant angular differences) with the factors of *Distance* (20 vs. 80 cm) and *Hesitation* (Mutual vs. Non-mutual). This analysis was intended to provide a time-averaged summary of pre-avoidance kinematic differences across the full one-second window, rather than to capture temporally localized effects. Temporally localized effects shortly before avoidance onset were more directly addressed by the Statistical Parametric Mapping (SPM) and linear mixed-effects models (LMM) analyses (see next two paragraphs).

#### Temporal changes of inter-participant angle difference

To identify the time regions where pre-avoidance kinematic differences appeared between mutual hesitation trials and non-mutual hesitation trials, we applied one-dimensional Statistical Parametric Mapping (SPM) analysis to the time-series data. The time window was from one second before the entry into the collision-risk zone, which was defined as the earlier onset of the two participants. At each time point, we tested for differences between mutual hesitation and non-mutual hesitation trials using SPM{t} (two-sample t-tests for independent groups), and then identified clusters of consecutive time points where the test statistic exceeded the critical threshold *z* (z*). These clusters were evaluated using random field theory to control the family-wise error rate at α = 0.05. SPM analysis was performed in MATLAB using the open-source spm1D toolbox (version 0.4.12; https://spm1d.org/).

#### Predicting the degree of mutual hesitation

To examine whether pre-avoidance kinematics predict the synchronization level of mutual hesitation, we fitted linear mixed-effects models (LMM) with RR as the dependent variable. We divided the last second before avoidance onset into 0.1-s time windows and computed inter-participant angular differences for the head, shoulders, and pelvis within each bin. Thus, the LMM tested whether kinematic differences observed in each 0.1-s interval before avoidance onset predicted the overall RR during the subsequent reaction phase. Each inter-participant angular differences for each time bin were included as a fixed effect, and we accounted for inter-dyad variability by adding dyad as a random effect. Covariates were included as controls. Multiple comparisons were controlled using the Benjamini–Hochberg FDR correction (q = 0.05). All statistical analyses, including ANOVA, SPM, and LMM, were conducted in MATLAB R2024a (The MathWorks Inc., Natick, MA, USA).

## Results

### CRQA

**Fig. 4:**
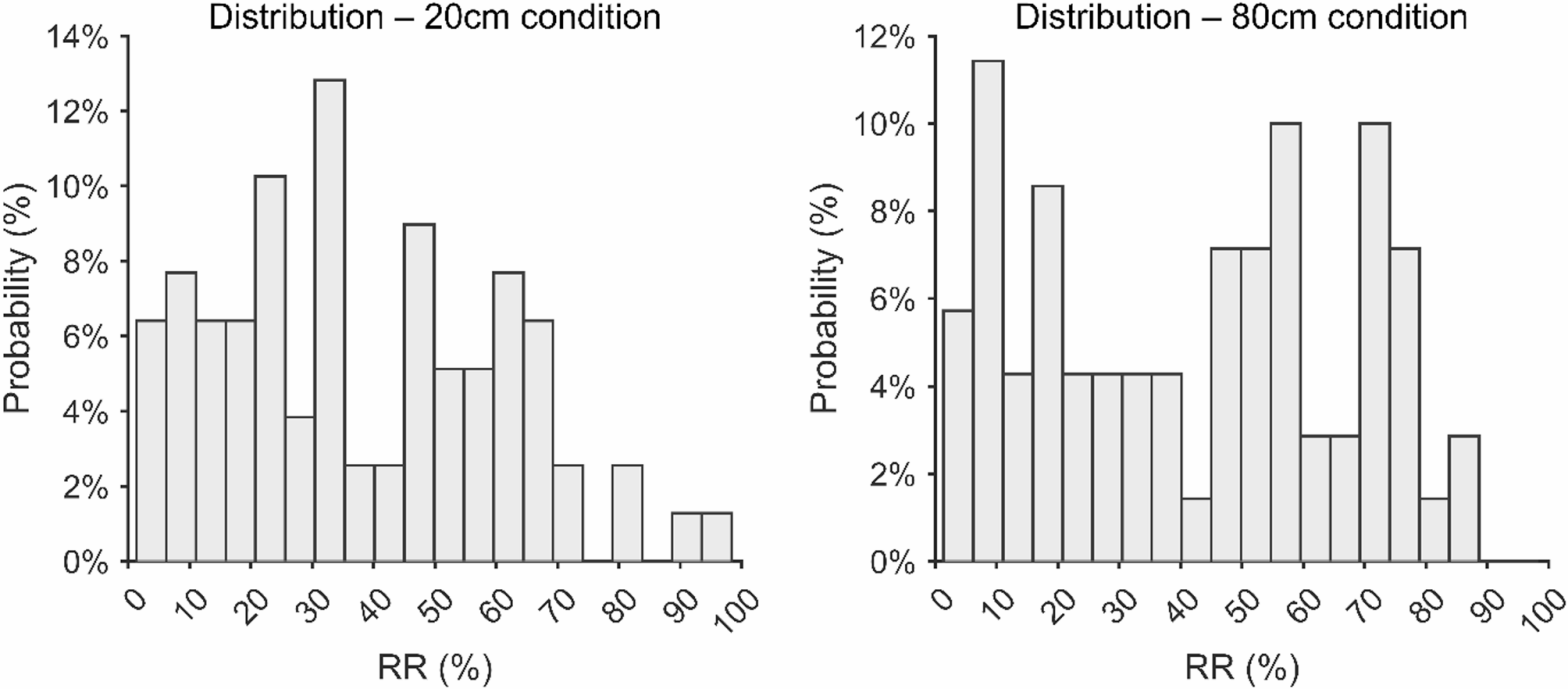
Distributions of recurrence rate (RR) in mutual hesitation trials for the 20 cm (left) and 80 cm (right) distance conditions.

In the 20 cm condition, 78 mutual hesitation events (9.2%) and 771 non-mutual hesitation events were observed. In the 80 cm condition, 70 mutual hesitation events (8.3%) and 774 non-mutual hesitation events were observed. Figure 4 shows the histograms of RR. The mean (SD) of RR in mutual hesitation trials was 37.97 (± 23.2) in the 20 cm condition and 41.95 (± 25.5) in the 80 cm condition.

#### Pre-avoidance kinematics between mutual hesitation and non-mutual hesitation

**Fig. 5:**
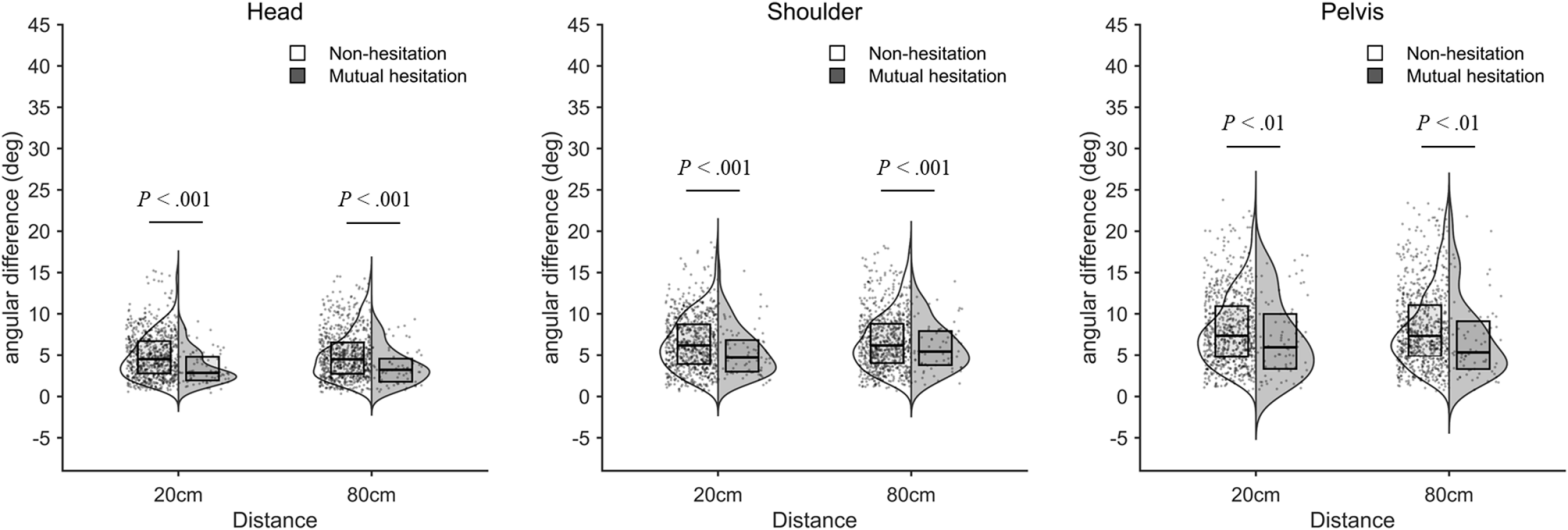
Inter-participant angular difference in the mutual hesitation (gray) and non-mutual hesitation (white) trials. Violin plots show the distribution; boxes indicate the median and interquartile range (IQR). Dots indicate individual data points.

Figure 5 presents the results of the two-way ANOVA. For the inter-participant head angle difference, a main effect of *Hesitation* was observed (F (1, 1689) = 60.42, *p* < .001, partial η² = 0.03), indicating that the presence of mutual hesitation showed smaller angle differences than non–mutual hesitation. The main effect of *Distance* was not significant (F (1, 1689) = 0.21, *p* = .65, partial η² = 0.00), nor was the *Distance* × *Hesitation* interaction (F (1, 1689) = 0.07, *p* = .79, partial η² = 0.00). For the inter-participant shoulder angle difference, the main effects of *Hesitation* (F (1, 1689) = 13.56, *p* < .001, partial η² = 0.01) were observed. The main effect of *Distance* was not significant (F (1, 1689) = 2.60, *p* = .11, partial η² = 0.00), nor was the *Distance* × *Hesitation* interaction (F (1, 1689) = 0.47, *p* = .50, partial η² = 0.000). For the inter-participant pelvis angle difference, the main effect of *Hesitation* (F (1, 1689) = 9.83, *p* < .01, partial η² = 0.01) was significant. The main effect of *Distance* was not significant (F (1, 1689) = 0.03, *p* = .86, partial η² = 0.00), nor the *Distance* × *Hesitation* interaction (F (1, 1689) = 0.12, *p* = .73, partial η² = 0.00).

#### Temporal changes of inter-participant angle difference

**Fig. 6:**
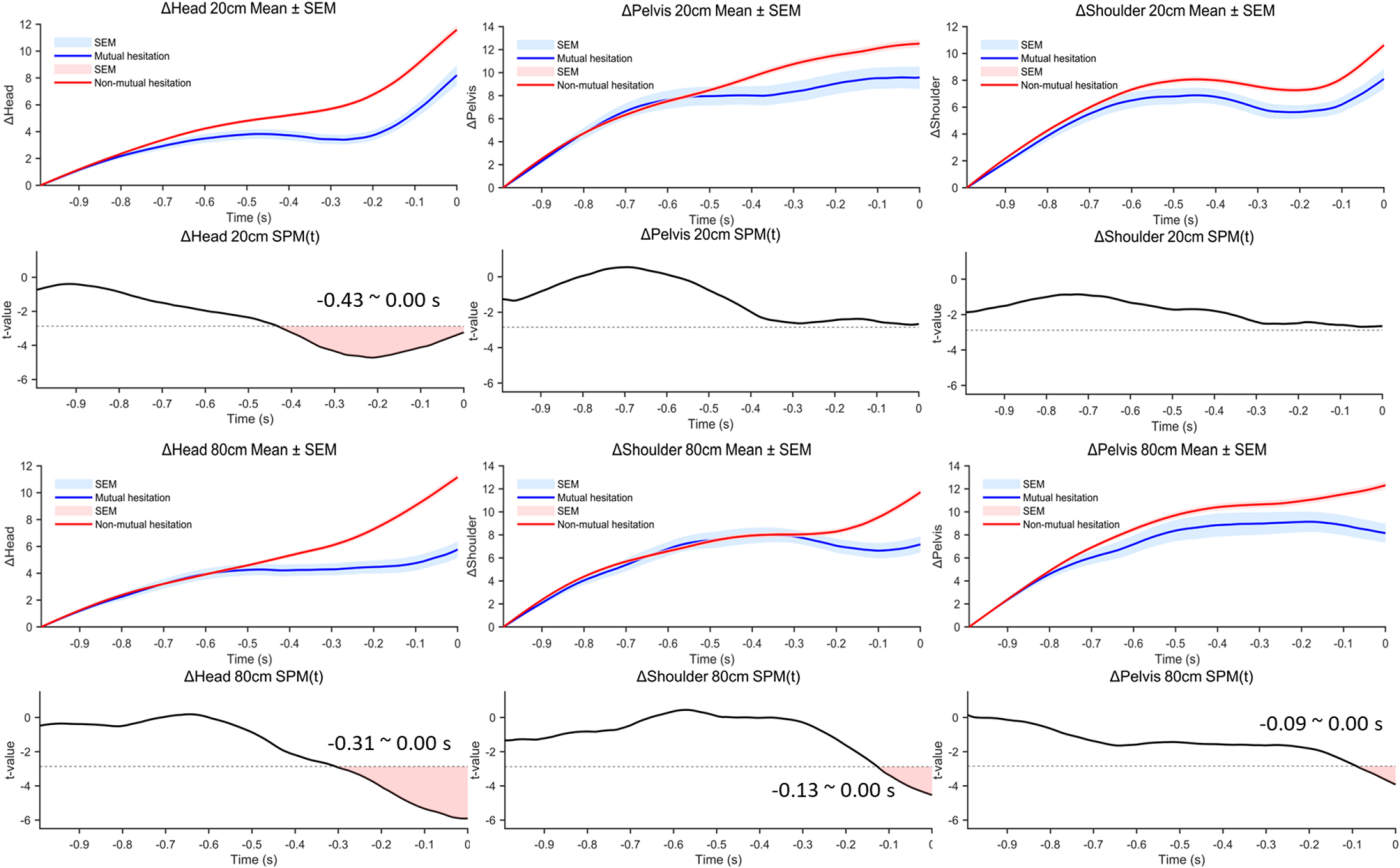
SPM results for inter-participant angular differences in the mutual hesitation and non-mutual hesitation trials. Top panels: mean ± SEM of ΔHead (left), ΔShoulder (middle), and ΔPelvis (right) over time. Bottom panels: time-varying t-statistics (SPM{t}) from the SPM analysis comparing the two conditions. The shaded cluster indicates the time interval in which a significant difference between conditions was detected.

Figure 6 shows the results of the SPM analysis. Under the 20 cm condition, a significant cluster was observed for the inter-participant head angle difference with a range of − 0.43 to 0 s (*p* < .001, peak *t* = 4.72, critical threshold z* = 2.87). No supra-threshold clusters were detected for the inter-participant shoulder or pelvis angle differences under the 20 cm condition. Under the 80 cm condition, significant clusters were observed for the inter-participant head angle difference (− 0.31 to 0 s, *p* < .001, peak *t* = 5.90; critical threshold z* = 2.86), the inter-participant shoulder angle difference (− 0.13 to 0 s, *p* < .01, peak *t* = 4.53, critical threshold z* = 2.87), and the inter-participant pelvis angle difference (− 0.09 to 0 s, *p* = .03, peak *t* = 3.91, critical threshold z* = 2.85). No additional supra-threshold clusters were identified. As a window-sensitivity analysis, we repeated the SPM analysis using a longer 2-s pre-avoidance window. The qualitative pattern of results was unchanged: significant differences were restricted to the final pre-avoidance interval only.

#### Predicting the degree of mutual hesitation

**Fig. 7:**
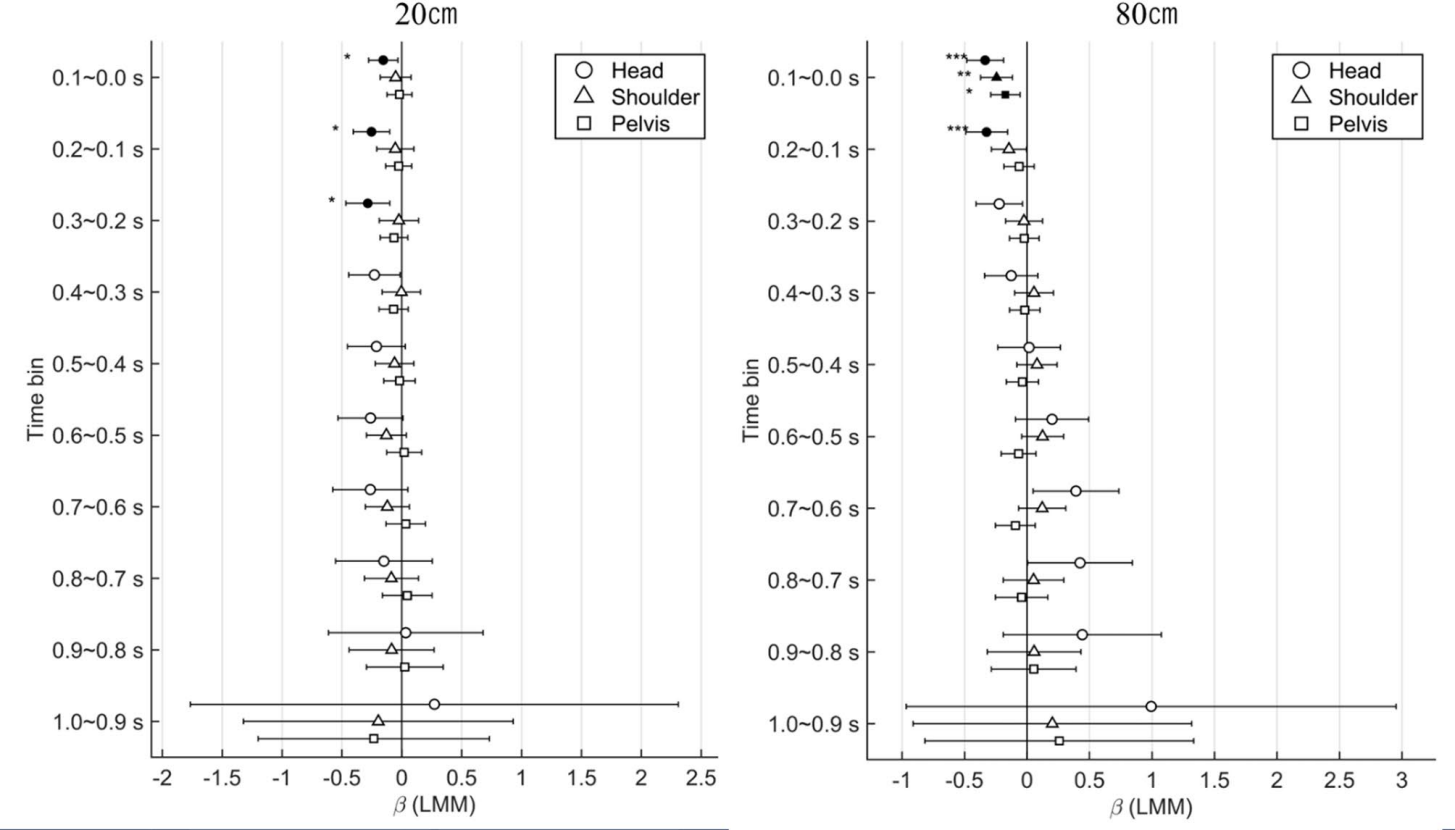
Forest plots of LMM fixed-effect estimates (β) for the relationship between inter-participant angular differences and RR (synchrony level). Circles, triangles, and squares represent the head, shoulder, and pelvis, respectively. The horizontal bars indicate 95% confidence intervals, and filled markers indicate significant effects. Asterisks indicate effects that remained significant after FDR correction (* q < 0.05, ** q < 0.01, *** q < 0.001). Exact p-values, FDR-corrected q-values, and the number of observations included in each model are reported in Supplementary Table S2.

Figure 7 shows the results of the LMM analysis. For the 20 cm condition, head-angle differences significantly predicted RR in three late time bins: 0.3–0.2 s (β = −0.28, 95% CI [− 0.47, − 0.10], q = 0.01), 0.2–0.1 s (β = −0.25, 95% CI [− 0.41, − 0.10], q = 0.01), and 0.1–0 s (β = −0.16, 95% CI [− 0.28, − 0.03], q = 0.04). Shoulder and pelvis-angle differences did not reach significance in any bin. For the 80 cm condition, head-angle differences were robust predictors, especially in later bins: 0.2–0.1 (β = −0.32, 95% CI [− 0.49, − 0.16], q < 0.001) and 0.1–0 (β = −0.34, 95% CI [− 0.48, − 0.19], q < 0.001). Shoulder-angle differences at 80 cm were significant in the 0.1–0 bin (β = −0.24, 95% CI [− 0.37, − 0.12], q < 0.01). Pelvis-angle differences at 80 cm were also significant in the 0.1–0 bin (β = −0.17, 95% CI [− 0.29, − 0.06], q = 0.04). All significant coefficients were negative, indicating that larger dyadic angle divergence in these windows were associated with lower levels of mutual hesitation. Detailed statistics for all bins are provided in the Supplementary Material, Table 2.

## Discussion

In this study, we quantified mutual hesitation by using CRQA and identified kinematic predictors of mutual hesitation, such as the inter-participant head angular difference, in near-collision avoidance scenarios. Accordingly, we used non-mutual-hesitation trials as a control or comparison condition to isolate kinematic features specifically associated with mutual hesitation. Our results show that (i) mutual hesitation was more likely when inter-participant angular differences—especially in head rotation—were small (i.e., the participant’s head orientations were aligned); (ii) in trials without mutual hesitation, inter-participant head-angle diverged earlier than in mutual hesitation trials; and (iii) inter-participant head-angle difference was the primary predictor of the degree of mutual hesitation. Together, these results indicate that inferring action intentions (i.e., walking direction) from head kinematics is critical for mutual anticipation and successful collision avoidance.

Before we delve into the discussion of the main findings, we aim to stress the advantages of CRQA as a novel and parsimonious method to quantify and illustrate the degree of mutual hesitation (i.e., spatiotemporal overlap between walkers) in near-collision avoidance (see Fig. 2). Previous trajectory-based approaches already convincingly indicated that mutual hesitation arises from dynamic interpersonal coupling, rather than from isolated individual decisions^1^. Yet, such approaches do not allow for capturing how this coupling evolves as a coordinated dynamical process within an interaction, nor how moment-to-moment dependencies scale into global behavioral patterns. CRQA resolves this limitation by transforming two time series (like two walkers’ trajectories) into one representation of the underlying dynamic structure, revealing recurrent patterns, coupling strengths, and coordination breakdowns^23,41,53^. Grounded in nonlinear dynamical-systems theory, CRQA offers a tool for characterizing how two walkers’ movements continuously co-evolve, directly addressing the interpersonal dynamical mechanisms that explain why mutual hesitation emerges and to what degree it unfolds. In addition, as illustrated in Fig. 2, CRQA allows us to visualize and quantify the dyadic interaction between walkers within a single framework^54^, including lagged coordination and leader–follower relations. More specifically, in a cross-recurrence plot (CRP; see Fig. 2), which visually shows the synchrony of two time series data, the diagonal line indicates a matching pattern, while the horizontal or vertical offset from the diagonal line represents who is leading or following, allowing us to visually capture the dyadic pattern, including time delayed interaction. While trajectory analysis can assess hesitation (e.g., the amount of time or distance two participants are moving in the same direction), it assumes direction changes and may miss pre-turn hesitation, such as brief deceleration or stalling. In other words, quantifying mutual hesitation—including the stagnation between the two pedestrians—may be difficult by only analyzing trajectories, and it seems more promising to detect mutual hesitation with CRQA. In this vein, when we compared the detection of overlap based on trajectory analysis (i.e., potential mutual hesitation) with the detection of mutual hesitation based on CRQA, the CRQA-based classification identified a larger number of mutual hesitation trials (see supplementary information, Table 3). Moreover, CRQA allows us to capture different forms of hesitation within a single framework, which is challenging when analyzing only trajectories. For instance, when walkers change direction back and forth several times, it is necessary to decide the definitions, such as where one turn ends and the next begins. Instead, CRQA treats the avoidance behaviors as one coupled dynamical system. Then, the corresponding CRP shows how the movements match, lag, or stall between each other, and quantifies how prolonged and irregular this repetitive hesitation is in a single evaluation frame. Taken together, we emphasize that CRQA is particularly useful for quantifying mutual hesitation in near-collision avoidance scenarios. In future research, this dyadic CRQA-based approach could be extended to other two-person interaction geometries, such as oblique approaches, by applying it to task-relevant paired kinematic time series, such as lateral position, heading direction, or movement velocity. Because CRQA is primarily designed to quantify recurrence between two time series^55^, extending the present approach to multi-agent collision avoidance would require further methodological development. Related recurrence-based approaches, such as multidimensional recurrence quantification analysis, have been proposed for analyzing multiple time series and group-level joint action^56^, but future work should clarify how such methods can be adapted to multi-agent collision avoidance.

Turning to the identification of kinematic predictors of mutual hesitation, SPM analysis showed that, in both 20 cm and 80 cm conditions, the earliest significant difference between the mutual hesitation trials and non-mutual hesitation trials appeared in the inter-participant head angle (0.43s before the avoidance onset for the 20 cm condition and 0.31s before the avoidance onset for the 80 cm condition). That is, it was revealed that the inter-participant head angle diverged earlier in the non-mutual hesitation trial than in mutual hesitation trials. In addition, LMM revealed that inter-participant head-angle difference was the earliest and most consistent predictor of RR, indicating that a smaller head angle difference (i.e., more direct facing between participants) was significantly associated with a greater degree of hesitation, whereas shoulder- and pelvis-angle differences also predicted RR in the final pre-avoidance bin under the 80 cm condition. Crucially, the effect remained significant when we controlled for external environmental covariates that have been reported in previous work (entry timing, ML clearance at entry, step-side, and mean walking speed, and inter-participant walking-speed difference). Our results add to previous work highlighting the importance of kinematic cues in mutual anticipation^52^, by showing that in collision avoidance scenarios mutual anticipation informed by head kinematics (and inter-participant head angle differences) is essential for successful dyadic coordination (i.e., successful collision avoidance).

As a possible conceptual interpretation, the framework of active-inference^57–59^ may be helpful to explain the present results at a computational level. Collision avoidance is, so to speak, ‘an online mutual-inference task^60^’, in which both pedestrians are required to recursively simulate their own future behavior and make a decision (e.g., avoiding left or right, decreasing the speed, or stopping) in an uncertain and unstable situation. From this perspective, if both participants face each other directly until the very last moment without an explicit intention of avoidance direction, they would engage in deeper recursive mutual reasoning (e.g., I may go left, but I may also go right based on his movement and so forth), thereby leading to delayed decision-making or indecision. Conversely, if the difference in the inter-participant head angle begins to diverge early, the prior distribution regarding the other participant’s behavior rapidly converges, enabling the formation of the mutual coordinated strategy and smooth collision avoidance. Importantly, this active-inference account should be regarded as a potential conceptual interpretation of the present behavioral findings, rather than as a mechanism directly tested by our data.

Notably, the degrees of mutual hesitation between the 20 cm and 80 cm were almost the same. Results of SPM and LMM, however, showed significant differences not only in the head but also in the shoulder and pelvis angle differences under the 80 cm condition. Although the present study identifies head-angle differences as the earliest and most consistent predictor of mutual hesitation, the significant shoulder and pelvis effects in the 80 cm condition suggest that these segments may also contribute to avoidance behavior, particularly when participants have more time and space to anticipate each other’s actions. Taken together, when the time to anticipate the other’s actions within the collision avoidance zone was longer, hesitation was more likely to be evident in the whole-body kinematics. However, because the 80 cm condition affords shallower avoidance angles considering the distance between participants at the moment of avoidance, it basically requires less body rotation to avoid each other. In addition, the longer time-to-collision in the 80 cm condition provides greater temporal flexibility, allowing participants to decelerate while waiting and seeing rather than committing to an early body rotation. Clearly, more research is needed to determine how interpersonal distance affects mutual hesitation.

To test whether head kinematics play a causal role in mutual hesitation, future studies are advised to experimentally manipulate the availability and reliability of head-related cues. For example, researchers could partially occlude the other person’s head orientation while keeping the rest of the body visible. In immersive VR or avatar-based environments, head orientation can also be systematically perturbed to examine whether disrupting early head cues increases mutual hesitation and changes whole-body alignment. Such causal tests would clarify whether signaling intentions by means of head orientations reduces mutual hesitation under different environmental constraints.

Ultimately, a number of limitations of the present study deserve to be discussed. First, gaze behavior was not directly analyzed in the present study. Although we focused on full-body kinematics based on previous evidence that bodily cues can support mutual anticipation even without mutual gaze, gaze remains a central cue in interpersonal coordination^10^. Future studies could combine eye-tracking with full-body motion capture to examine how gaze and body kinematics jointly contribute to mutual hesitation. Second, because the same dyads interacted repeatedly, participants may have adapted to their partner’s behavior, developed implicit leader–follower roles, or adopted preferred avoidance strategies across trials. As an exploratory check (see the Supplementary Material, Fig. 2), we examined the trial-wise proportion of mutual hesitation across repeated trials, which showed no clear monotonic increase or decrease. However, dyad-specific adaptation cannot be ruled out completely. Future studies should vary interaction partners, randomize partner pairings, or include trial order as a planned factor to examine repetition effects more directly. Finally, CRQA parameters were calibrated using the additional experimental condition (instruction condition), in which avoidance directions were externally specified. Therefore, differences between instructed and spontaneous avoidance behavior may have influenced parameter generalizability. However, this condition was used only for parameter estimation, and all hypothesis testing was conducted on the main free-choice trials.

To conclude, in this study we examined mutual anticipation and the emergence and solution of near-collisions in truly interactive collision avoidance scenarios. We introduced cross-recurrence quantification analysis (CRQA) as a novel method to quantify the degree of mutual hesitation (i.e., spatiotemporal overlap between walkers) in near-collision avoidance. Our results showed that inter-participant head-angle differences diverged earlier than the shoulder or pelvis in non-mutual hesitation trials. Most importantly, smaller head-angle differences before avoidance onset predicted a higher degree of mutual hesitation. We therefore conclude that smaller inter-participant head-angle differences before avoidance onset are predictive of a higher degree of mutual hesitation, suggesting that failures in inferring avoidance direction from head kinematics may be linked to mutual anticipation errors in near-collision avoidance.

## Supplementary Information

Below is the link to the electronic supplementary material.

Supplementary Material 1

## Data Availability

The datasets analyzed during the current study are available from the corresponding author on reasonable request.
