# Supplementary material for: Quantifying mutual hesitation and identifying kinematic predictors in near-collision avoidance in walking
Source: Sci Rep. 2026 Jul 7;16:21400. doi: 10.1038/s41598-026-59801-3 (PMC13350750; doi:10.1038/s41598-026-59801-3)
Supplement: Supplementary file 1 — Supplementary Material 1 [file 41598_2026_59801_MOESM1_ESM.docx]

**Supplementary Material**


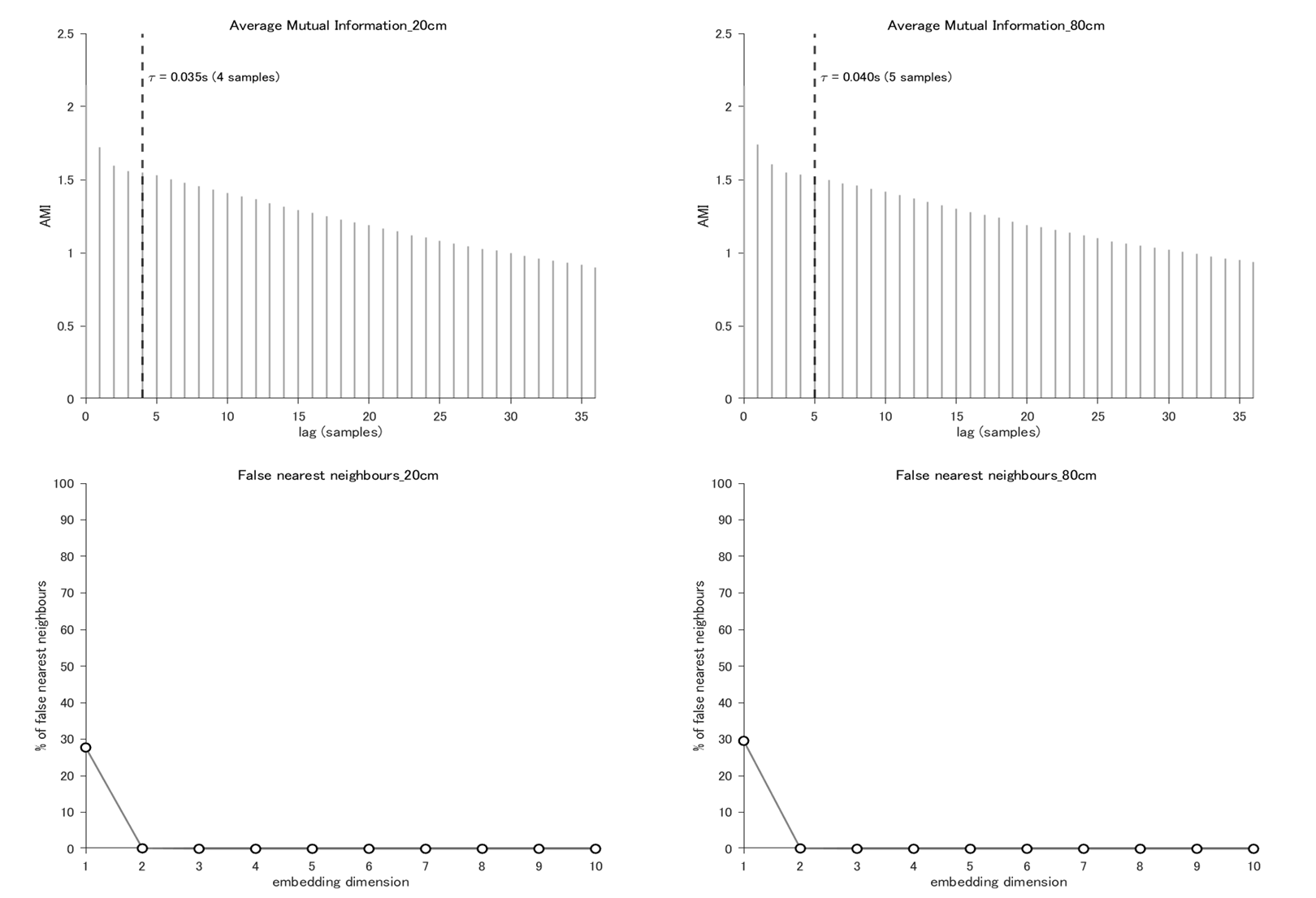


**Figure S1.** Average mutual information (AMI; top) and percentage of false nearest neighbors (FNN; bottom). The vertical dashed lines indicate the selected time delay (first local minimum of AMI). The proportion of false nearest neighbors drops to zero at an embedding dimension of m = 2, which was therefore chosen as the optimal embedding dimension.


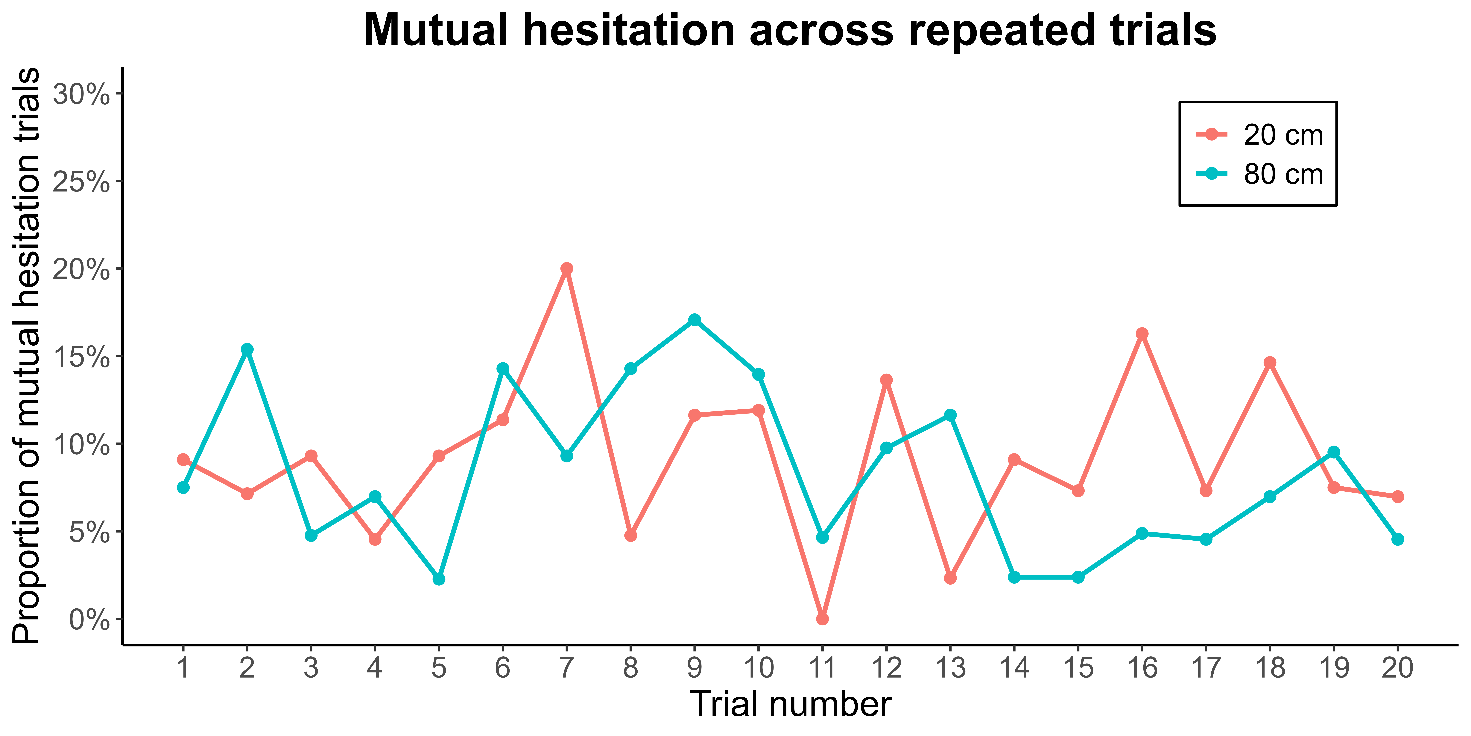


**Figure S2.** Trial-wise proportion of mutual hesitation across repeated trials. The proportion of trials classified as mutual hesitation is shown separately for the 20 cm and 80 cm collision avoidance zones.

**Table S1. The result of radius sweep**

| Distance | Instruction | Radius | TP | FP | FN | TN | N | MCC |
| --- | --- | --- | --- | --- | --- | --- | --- | --- |
| 20cm | instruction | 4.3 | 611 | 13 | 13 | 1092 | 1729 | 0.967401961 |
| 20cm | instruction | 4.4 | 611 | 13 | 13 | 1092 | 1729 | 0.967401961 |
| 20cm | instruction | 4.5 | 611 | 13 | 13 | 1092 | 1729 | 0.967401961 |
| 20cm | instruction | 4.6 | 611 | 13 | 13 | 1092 | 1729 | 0.967401961 |
| 20cm | instruction | 4.7 | 611 | 13 | 13 | 1092 | 1729 | 0.967401961 |
| 20cm | instruction | 4.8 | 611 | 13 | 13 | 1092 | 1729 | 0.967401961 |
| 20cm | instruction | 4.9 | 611 | 13 | 13 | 1092 | 1729 | 0.967401961 |
| 20cm | instruction | 5 | 611 | 13 | 13 | 1092 | 1729 | 0.967401961 |
| 20cm | instruction | 3.9 | 610 | 13 | 14 | 1092 | 1729 | 0.966137139 |
| 20cm | instruction | 4 | 610 | 13 | 14 | 1092 | 1729 | 0.966137139 |
| 20cm | instruction | 4.1 | 610 | 13 | 14 | 1092 | 1729 | 0.966137139 |
| 20cm | instruction | 4.2 | 610 | 13 | 14 | 1092 | 1729 | 0.966137139 |
| 20cm | instruction | 3.8 | 609 | 13 | 15 | 1092 | 1729 | 0.964872951 |
| 20cm | instruction | 3.7 | 608 | 13 | 16 | 1092 | 1729 | 0.963609394 |
| 20cm | instruction | 3.3 | 607 | 13 | 17 | 1092 | 1729 | 0.962346464 |
| 20cm | instruction | 3.4 | 607 | 13 | 17 | 1092 | 1729 | 0.962346464 |
| 20cm | instruction | 3.5 | 607 | 13 | 17 | 1092 | 1729 | 0.962346464 |
| 20cm | instruction | 3.6 | 607 | 13 | 17 | 1092 | 1729 | 0.962346464 |
| 20cm | instruction | 2.8 | 605 | 11 | 19 | 1094 | 1729 | 0.96233031 |
| 20cm | instruction | 2.9 | 605 | 11 | 19 | 1094 | 1729 | 0.96233031 |
| 20cm | instruction | 3.2 | 606 | 13 | 18 | 1092 | 1729 | 0.961084157 |
| 20cm | instruction | 3 | 605 | 12 | 19 | 1093 | 1729 | 0.961075175 |
| 20cm | instruction | 3.1 | 605 | 12 | 19 | 1093 | 1729 | 0.961075175 |
| 20cm | instruction | 2.5 | 601 | 10 | 23 | 1095 | 1729 | 0.958565603 |
| 20cm | instruction | 2.6 | 602 | 11 | 22 | 1094 | 1729 | 0.958558081 |
| 20cm | instruction | 2.7 | 602 | 11 | 22 | 1094 | 1729 | 0.958558081 |
| 20cm | instruction | 2 | 590 | 6 | 34 | 1099 | 1729 | 0.949951708 |
| 20cm | instruction | 2.3 | 593 | 9 | 31 | 1096 | 1729 | 0.949828369 |
| 20cm | instruction | 2.4 | 594 | 10 | 30 | 1095 | 1729 | 0.949799731 |
| 20cm | instruction | 2.1 | 590 | 7 | 34 | 1098 | 1729 | 0.948660801 |
| 20cm | instruction | 2.2 | 591 | 8 | 33 | 1097 | 1729 | 0.948617561 |
| 20cm | instruction | 1.9 | 587 | 6 | 37 | 1099 | 1729 | 0.946229252 |
| 20cm | instruction | 1.8 | 584 | 6 | 40 | 1099 | 1729 | 0.942511825 |
| 20cm | instruction | 1.7 | 579 | 6 | 45 | 1099 | 1729 | 0.93632703 |
| 20cm | instruction | 1.4 | 576 | 4 | 48 | 1101 | 1729 | 0.935256753 |
| 20cm | instruction | 1.6 | 578 | 6 | 46 | 1099 | 1729 | 0.935091676 |
| 20cm | instruction | 1.3 | 574 | 4 | 50 | 1101 | 1729 | 0.932796127 |
| 20cm | instruction | 1.5 | 576 | 6 | 48 | 1099 | 1729 | 0.932622545 |
| 20cm | instruction | 1.2 | 571 | 4 | 53 | 1101 | 1729 | 0.929109048 |
| 20cm | instruction | 1.1 | 567 | 4 | 57 | 1101 | 1729 | 0.924200001 |
| 20cm | instruction | 1 | 562 | 4 | 62 | 1101 | 1729 | 0.918074717 |
| 20cm | instruction | 0.9 | 556 | 4 | 68 | 1101 | 1729 | 0.910739964 |
| 20cm | instruction | 0.8 | 552 | 4 | 72 | 1101 | 1729 | 0.905859222 |
| 20cm | instruction | 0.7 | 547 | 4 | 77 | 1101 | 1729 | 0.899768125 |
| 20cm | instruction | 0.6 | 543 | 4 | 81 | 1101 | 1729 | 0.894902836 |
| 20cm | instruction | 0.5 | 538 | 4 | 86 | 1101 | 1729 | 0.888830335 |
| 20cm | instruction | 0.4 | 531 | 3 | 93 | 1102 | 1729 | 0.881740894 |
| 20cm | instruction | 0.3 | 521 | 3 | 103 | 1102 | 1729 | 0.869666857 |
| 20cm | instruction | 0.2 | 497 | 1 | 127 | 1104 | 1729 | 0.843738364 |
| 20cm | instruction | 0.1 | 441 | 1 | 183 | 1104 | 1729 | 0.777088621 |
| 80cm | instruction | 2.1 | 696 | 24 | 17 | 997 | 1734 | 0.951275055 |
| 80cm | instruction | 2.2 | 696 | 24 | 17 | 997 | 1734 | 0.951275055 |
| 80cm | instruction | 2 | 694 | 22 | 19 | 999 | 1734 | 0.951206926 |
| 80cm | instruction | 3.7 | 705 | 34 | 8 | 987 | 1734 | 0.950703464 |
| 80cm | instruction | 3.8 | 705 | 34 | 8 | 987 | 1734 | 0.950703464 |
| 80cm | instruction | 3.9 | 705 | 34 | 8 | 987 | 1734 | 0.950703464 |
| 80cm | instruction | 4 | 705 | 34 | 8 | 987 | 1734 | 0.950703464 |
| 80cm | instruction | 4.1 | 705 | 34 | 8 | 987 | 1734 | 0.950703464 |
| 80cm | instruction | 4.2 | 705 | 34 | 8 | 987 | 1734 | 0.950703464 |
| 80cm | instruction | 4.3 | 705 | 34 | 8 | 987 | 1734 | 0.950703464 |
| 80cm | instruction | 4.4 | 705 | 34 | 8 | 987 | 1734 | 0.950703464 |
| 80cm | instruction | 4.5 | 705 | 34 | 8 | 987 | 1734 | 0.950703464 |
| 80cm | instruction | 4.6 | 705 | 34 | 8 | 987 | 1734 | 0.950703464 |
| 80cm | instruction | 4.7 | 705 | 34 | 8 | 987 | 1734 | 0.950703464 |
| 80cm | instruction | 4.8 | 705 | 34 | 8 | 987 | 1734 | 0.950703464 |
| 80cm | instruction | 4.9 | 705 | 34 | 8 | 987 | 1734 | 0.950703464 |
| 80cm | instruction | 5 | 705 | 34 | 8 | 987 | 1734 | 0.950703464 |
| 80cm | instruction | 1.9 | 693 | 22 | 20 | 999 | 1734 | 0.950002761 |
| 80cm | instruction | 1.8 | 692 | 21 | 21 | 1000 | 1734 | 0.949978914 |
| 80cm | instruction | 3.1 | 704 | 34 | 9 | 987 | 1734 | 0.949473892 |
| 80cm | instruction | 3.2 | 704 | 34 | 9 | 987 | 1734 | 0.949473892 |
| 80cm | instruction | 3.3 | 704 | 34 | 9 | 987 | 1734 | 0.949473892 |
| 80cm | instruction | 3.4 | 704 | 34 | 9 | 987 | 1734 | 0.949473892 |
| 80cm | instruction | 3.5 | 704 | 34 | 9 | 987 | 1734 | 0.949473892 |
| 80cm | instruction | 3.6 | 704 | 34 | 9 | 987 | 1734 | 0.949473892 |
| 80cm | instruction | 2.8 | 703 | 33 | 10 | 988 | 1734 | 0.949388716 |
| 80cm | instruction | 2.9 | 703 | 33 | 10 | 988 | 1734 | 0.949388716 |
| 80cm | instruction | 3 | 703 | 33 | 10 | 988 | 1734 | 0.949388716 |
| 80cm | instruction | 2.5 | 699 | 29 | 14 | 992 | 1734 | 0.949100434 |
| 80cm | instruction | 2.7 | 701 | 32 | 12 | 989 | 1734 | 0.948084471 |
| 80cm | instruction | 2.6 | 700 | 31 | 13 | 990 | 1734 | 0.948011957 |
| 80cm | instruction | 1.7 | 690 | 21 | 23 | 1000 | 1734 | 0.947577467 |
| 80cm | instruction | 2.4 | 697 | 29 | 16 | 992 | 1734 | 0.946665814 |
| 80cm | instruction | 2.3 | 696 | 28 | 17 | 993 | 1734 | 0.946611264 |
| 80cm | instruction | 1.6 | 687 | 20 | 26 | 1001 | 1734 | 0.945169569 |
| 80cm | instruction | 1.4 | 686 | 19 | 27 | 1002 | 1734 | 0.945165175 |
| 80cm | instruction | 1.5 | 686 | 19 | 27 | 1002 | 1734 | 0.945165175 |
| 80cm | instruction | 1.3 | 684 | 18 | 29 | 1003 | 1734 | 0.943974851 |
| 80cm | instruction | 1.2 | 680 | 18 | 33 | 1003 | 1734 | 0.939217599 |
| 80cm | instruction | 1 | 677 | 16 | 36 | 1005 | 1734 | 0.938074416 |
| 80cm | instruction | 1.1 | 677 | 16 | 36 | 1005 | 1734 | 0.938074416 |
| 80cm | instruction | 0.9 | 675 | 15 | 38 | 1006 | 1734 | 0.936924552 |
| 80cm | instruction | 0.8 | 673 | 15 | 40 | 1006 | 1734 | 0.934566694 |
| 80cm | instruction | 0.7 | 664 | 15 | 49 | 1006 | 1734 | 0.923994059 |
| 80cm | instruction | 0.6 | 657 | 13 | 56 | 1008 | 1734 | 0.918295338 |
| 80cm | instruction | 0.5 | 650 | 11 | 63 | 1010 | 1734 | 0.912678503 |
| 80cm | instruction | 0.4 | 641 | 9 | 72 | 1012 | 1734 | 0.904847808 |
| 80cm | instruction | 0.3 | 626 | 4 | 87 | 1017 | 1734 | 0.894222062 |
| 80cm | instruction | 0.2 | 591 | 2 | 122 | 1019 | 1734 | 0.857744348 |
| 80cm | instruction | 0.1 | 514 | 0 | 199 | 1021 | 1734 | 0.776729411 |
| Note: TP (true positives) and TN (true negatives) show trials that were correctly classified as the presence or absence of overlap/mutual hesitation, whereas FP (false positives) and FN (false negatives) denote misclassified trials. | | | | | | | | |

**Table S2. The results of LMM**

| Head_20cm | | | | | | | | |
| --- | --- | --- | --- | --- | --- | --- | --- | --- |
| Time Bin | N | Beta | SE | t-value | p-value | q-FDR | CI95_L | CI95_U |
| Head_20cm_1.0~0.9 | 849 | 0.27 | 1.04 | 0.26 | 0.79 | 0.88 | -1.77 | 2.31 |
| Head_20cm_0.9~0.8 | 849 | 0.03 | 0.33 | 0.10 | 0.92 | 0.92 | -0.61 | 0.68 |
| Head_20cm_0.8~0.7 | 849 | -0.15 | 0.21 | -0.73 | 0.47 | 0.58 | -0.55 | 0.25 |
| Head_20cm_0.7~0.6 | 849 | -0.26 | 0.16 | -1.65 | 0.10 | 0.14 | -0.58 | 0.05 |
| Head_20cm_0.6~0.5 | 849 | -0.26 | 0.14 | -1.89 | 0.06 | 0.12 | -0.53 | 0.01 |
| Head_20cm_0.5~0.4 | 849 | -0.21 | 0.12 | -1.72 | 0.08 | 0.14 | -0.45 | 0.03 |
| Head_20cm_0.4~0.3 | 849 | -0.23 | 0.11 | -2.10 | 0.04 | 0.09 | -0.44 | -0.01 |
| Head_20cm_0.3~0.2 | 849 | -0.28 | 0.09 | -3.04 | 0.00 | 0.01 | -0.47 | -0.10 |
| Head_20cm_0.2~0.1 | 849 | -0.25 | 0.08 | -3.27 | 0.00 | 0.01 | -0.41 | -0.10 |
| Head_20cm_0.1~0.0 | 849 | -0.16 | 0.06 | -2.48 | 0.01 | 0.04 | -0.28 | -0.03 |
| Head_80cm | | | | | | | | |
| Head_80cm_1.0~0.9 | 844 | 0.99 | 1.00 | 1.00 | 0.32 | 0.36 | -0.97 | 2.95 |
| Head_80cm_0.9~0.8 | 844 | 0.44 | 0.32 | 1.38 | 0.17 | 0.26 | -0.19 | 1.07 |
| Head_80cm_0.8~0.7 | 844 | 0.42 | 0.21 | 1.99 | 0.05 | 0.09 | 0.00 | 0.84 |
| Head_80cm_0.7~0.6 | 844 | 0.39 | 0.17 | 2.24 | 0.03 | 0.06 | 0.05 | 0.73 |
| Head_80cm_0.6~0.5 | 844 | 0.20 | 0.15 | 1.35 | 0.18 | 0.26 | -0.09 | 0.49 |
| Head_80cm_0.5~0.4 | 844 | 0.02 | 0.13 | 0.13 | 0.90 | 0.90 | -0.23 | 0.27 |
| Head_80cm_0.4~0.3 | 844 | -0.13 | 0.11 | -1.17 | 0.24 | 0.30 | -0.34 | 0.09 |
| Head_80cm_0.3~0.2 | 844 | -0.22 | 0.10 | -2.34 | 0.02 | 0.06 | -0.41 | -0.04 |
| Head_80cm_0.2~0.1 | 844 | -0.32 | 0.09 | -3.80 | 0.00 | 0.00 | -0.49 | -0.16 |
| Head_80cm_0.1~0.0 | 844 | -0.34 | 0.08 | -4.46 | 0.00 | 0.00 | -0.48 | -0.19 |
| Shoulder_20cm | | | | | | | | |
| Shoulder_20cm_1.0~0.9 | 849 | -0.20 | 0.57 | -0.34 | 0.73 | 0.86 | -1.32 | 0.93 |
| Shoulder_20cm_0.9~0.8 | 849 | -0.08 | 0.18 | -0.47 | 0.64 | 0.86 | -0.44 | 0.27 |
| Shoulder_20cm_0.8~0.7 | 849 | -0.09 | 0.11 | -0.76 | 0.45 | 0.82 | -0.31 | 0.14 |
| Shoulder_20cm_0.7~0.6 | 849 | -0.12 | 0.09 | -1.29 | 0.20 | 0.82 | -0.31 | 0.06 |
| Shoulder_20cm_0.6~0.5 | 849 | -0.13 | 0.09 | -1.52 | 0.13 | 0.82 | -0.30 | 0.04 |
| Shoulder_20cm_0.5~0.4 | 849 | -0.06 | 0.08 | -0.74 | 0.46 | 0.82 | -0.22 | 0.10 |
| Shoulder_20cm_0.4~0.3 | 849 | 0.00 | 0.08 | -0.05 | 0.96 | 0.96 | -0.16 | 0.16 |
| Shoulder_20cm_0.3~0.2 | 849 | -0.02 | 0.08 | -0.29 | 0.77 | 0.86 | -0.19 | 0.14 |
| Shoulder_20cm_0.2~0.1 | 849 | -0.05 | 0.08 | -0.69 | 0.49 | 0.82 | -0.21 | 0.10 |
| Shoulder_20cm_0.1~0.0 | 849 | -0.05 | 0.07 | -0.79 | 0.43 | 0.82 | -0.18 | 0.08 |
| Shoulder_80cm | | | | | | | | |
| Shoulder_80cm_1.0~0.9 | 844 | 0.20 | 0.57 | 0.36 | 0.72 | 0.77 | -0.91 | 1.32 |
| Shoulder_80cm_0.9~0.8 | 844 | 0.06 | 0.19 | 0.30 | 0.77 | 0.77 | -0.32 | 0.43 |
| Shoulder_80cm_0.8~0.7 | 844 | 0.05 | 0.12 | 0.42 | 0.67 | 0.77 | -0.19 | 0.29 |
| Shoulder_80cm_0.7~0.6 | 844 | 0.12 | 0.10 | 1.26 | 0.21 | 0.52 | -0.07 | 0.31 |
| Shoulder_80cm_0.6~0.5 | 844 | 0.12 | 0.09 | 1.45 | 0.15 | 0.49 | -0.04 | 0.29 |
| Shoulder_80cm_0.5~0.4 | 844 | 0.08 | 0.08 | 0.98 | 0.33 | 0.66 | -0.08 | 0.24 |
| Shoulder_80cm_0.4~0.3 | 844 | 0.06 | 0.08 | 0.71 | 0.48 | 0.77 | -0.10 | 0.21 |
| Shoulder_80cm_0.3~0.2 | 844 | -0.02 | 0.08 | -0.32 | 0.75 | 0.77 | -0.17 | 0.12 |
| Shoulder_80cm_0.2~0.1 | 844 | -0.14 | 0.07 | -2.02 | 0.04 | 0.22 | -0.29 | 0.00 |
| Shoulder_80cm_0.1~0.0 | 844 | -0.24 | 0.07 | -3.75 | 0.00 | 0.00 | -0.37 | -0.12 |
| Pelvis_20cm | | | | | | | | |
| Pelvis_20cm_1.0~0.9 | 849 | -0.23 | 0.49 | -0.48 | 0.63 | 0.88 | -1.20 | 0.73 |
| Pelvis_20cm_0.9~0.8 | 849 | 0.02 | 0.16 | 0.15 | 0.88 | 0.88 | -0.30 | 0.34 |
| Pelvis_20cm_0.8~0.7 | 849 | 0.05 | 0.11 | 0.43 | 0.67 | 0.88 | -0.16 | 0.25 |
| Pelvis_20cm_0.7~0.6 | 849 | 0.03 | 0.08 | 0.39 | 0.70 | 0.88 | -0.13 | 0.20 |
| Pelvis_20cm_0.6~0.5 | 849 | 0.02 | 0.07 | 0.26 | 0.79 | 0.88 | -0.13 | 0.17 |
| Pelvis_20cm_0.5~0.4 | 849 | -0.02 | 0.07 | -0.29 | 0.77 | 0.88 | -0.15 | 0.11 |
| Pelvis_20cm_0.4~0.3 | 849 | -0.07 | 0.06 | -1.11 | 0.27 | 0.88 | -0.19 | 0.05 |
| Pelvis_20cm_0.3~0.2 | 849 | -0.07 | 0.06 | -1.12 | 0.26 | 0.88 | -0.18 | 0.05 |
| Pelvis_20cm_0.2~0.1 | 849 | -0.03 | 0.06 | -0.47 | 0.64 | 0.88 | -0.14 | 0.08 |
| Pelvis_20cm_0.1~0.0 | 849 | -0.02 | 0.05 | -0.37 | 0.71 | 0.88 | -0.12 | 0.08 |
| Pelvis_80cm | | | | | | | | |
| Pelvis_80cm_1.0~0.9 | 844 | 0.26 | 0.55 | 0.47 | 0.64 | 0.77 | -0.82 | 1.33 |
| Pelvis_80cm_0.9~0.8 | 844 | 0.05 | 0.17 | 0.31 | 0.76 | 0.77 | -0.29 | 0.39 |
| Pelvis_80cm_0.8~0.7 | 844 | -0.04 | 0.11 | -0.41 | 0.68 | 0.77 | -0.25 | 0.17 |
| Pelvis_80cm_0.7~0.6 | 844 | -0.09 | 0.08 | -1.15 | 0.25 | 0.77 | -0.25 | 0.07 |
| Pelvis_80cm_0.6~0.5 | 844 | -0.07 | 0.07 | -0.95 | 0.34 | 0.77 | -0.21 | 0.07 |
| Pelvis_80cm_0.5~0.4 | 844 | -0.04 | 0.07 | -0.57 | 0.57 | 0.77 | -0.17 | 0.09 |
| Pelvis_80cm_0.4~0.3 | 844 | -0.02 | 0.06 | -0.30 | 0.77 | 0.77 | -0.14 | 0.10 |
| Pelvis_80cm_0.3~0.2 | 844 | -0.02 | 0.06 | -0.38 | 0.71 | 0.77 | -0.14 | 0.10 |
| Pelvis_80cm_0.2~0.1 | 844 | -0.06 | 0.06 | -1.02 | 0.31 | 0.77 | -0.18 | 0.06 |
| Pelvis_80cm_0.1~0.0 | 844 | -0.17 | 0.06 | -2.90 | 0.00 | 0.04 | -0.29 | -0.06 |

**Table S3. The results of SPM**

| **Classification method** | **Distance** | **Metric** | **n_Positive** | **n_Negative** | **z* (threshold)** | **max \|t\|** | **Cluster range (idx)** | **Cluster range (s)** | **Cluster**  **p-value** |
| --- | --- | --- | --- | --- | --- | --- | --- | --- | --- |
| CRQA | 20 | ΔHead | 78 | 771 | 2.872 | 4.715 | 68–119 | -0.432 to 0.000 | 0 |
| CRQA | 20 | ΔShoulder | 78 | 771 | 2.878 | 2.688 | - | - | - |
| CRQA | 20 | ΔPelvis | 78 | 771 | 2.85 | 2.706 | - | - | - |
| CRQA | 80 | ΔHead | 70 | 774 | 2.859 | 5.903 | 83–119 | -0.305 to 0.000 | 0 |
| CRQA | 80 | ΔShoulder | 70 | 774 | 2.873 | 4.525 | 104–119 | -0.127 to 0.000 | 0.0102 |
| CRQA | 80 | ΔPelvis | 70 | 774 | 2.845 | 3.913 | 109–119 | -0.085 to 0.000 | 0.0257 |
| Trajectory analysis | 20 | ΔHead | 66 | 783 | 2.872 | 4.226 | 74–119 | -0.381 to 0.000 | 0 |
| Trajectory analysis | 20 | ΔShoulder | 66 | 783 | 2.877 | 2.08 | - | - | - |
| Trajectory analysis | 20 | ΔPelvis | 66 | 783 | 2.85 | 2.545 | - | - | - |
| Trajectory analysis | 80 | ΔHead | 70 | 774 | 2.86 | 5.319 | 90–119 | -0.246 to 0.000 | 0 |
| Trajectory analysis | 80 | ΔShoulder | 70 | 774 | 2.873 | 4.325 | 106–119 | -0.110 to 0.000 | 0.0144 |
| Trajectory analysis | 80 | ΔPelvis | 70 | 774 | 2.845 | 3.373 | 115–119 | -0.034 to 0.000 | 0.0445 |
| Note: Comparison of SPM results using CRQA-based and trajectory-based classifications. In the CRQA analysis, Positive indicates mutual hesitation trials; in the trajectory analysis, Positive indicates overlap trials (potential mutual hesitation trials). Cluster ranges indicate suprathreshold clusters identified by SPM{t}. Cluster p-values are random-field-theory-corrected p-values. | | | | | | | | | |

**Table S4. Distribution of excluded trials**

| **Distance (cm)** | **Initial trials after dyad exclusion** | **CRQA failure** | **Angular-difference outliers** | **Missing marker** | **Primary analysis N** | **Sensitivity analysis N** |
| --- | --- | --- | --- | --- | --- | --- |
| 20 cm | 880 | 14 | 17 | 0 | 849 | 866 |
| 80 cm | 880 | 20 | 15 | 1 | 844 | 859 |
| Note. Angular-difference outliers were retained only for the sensitivity analysis. CRQA-failure trials and the missing-marker trial could not be retained because valid RR values or reliable kinematics were unavailable. | | | | | | |

**Table S5. Sensitivity analysis: SPM results**

| **Distance (cm)** | **Metric** | **n_Positive** | **n_Negative** | **Cluster range (s)** | **Cluster p-value** |
| --- | --- | --- | --- | --- | --- |
| 20 cm | ΔHead | 80 | 786 | -0.395 to -0.025 | 0 |
| 20 cm | ΔShoulder | 80 | 786 | - | - |
| 20 cm | ΔPelvis | 80 | 786 | -0.319 to -0.269 | 0.034 |
| 80 cm | ΔHead | 71 | 788 | -0.294 to 0.000 | 0 |
| 80 cm | ΔShoulder | 71 | 788 | -0.126 to 0.000 | 0.009 |
| 80 cm | ΔPelvis | 71 | 788 | -0.118 to 0.000 | 0.015 |
| Note; SPM clusters were obtained from the sensitivity analysis in which angular-difference outlier trials were retained, but angular-difference values exceeding the predefined outlier thresholds were capped at the corresponding threshold: 15.5° for the head, 18.7° for the shoulder, and 24.0° for the pelvis. | | | | | |

**Table S6. Sensitivity analysis: LMM results**

| **Distance** | **Metric** | **Time bin** | **N** | **Beta** | **SE** | **t** | **p** | **q_FDR** |
| --- | --- | --- | --- | --- | --- | --- | --- | --- |
| 20 cm | Head | -1.0 to -0.9 s | 866 | 2.104 | 0.770 | 2.731 | 0.006 | 0.025 |
| 20 cm | Head | -0.3 to -0.2 s | 866 | -0.279 | 0.104 | -2.686 | 0.007 | 0.025 |
| 20 cm | Head | -0.2 to -0.1 s | 866 | -0.272 | 0.097 | -2.817 | 0.005 | 0.025 |
| 80 cm | Head | -0.8 to -0.7 s | 859 | 0.497 | 0.208 | 2.395 | 0.017 | 0.042 |
| 80 cm | Head | -0.7 to -0.6 s | 859 | 0.435 | 0.167 | 2.611 | 0.009 | 0.031 |
| 80 cm | Head | -0.2 to -0.1 s | 859 | -0.395 | 0.107 | -3.705 | 0.000 | 0.001 |
| 80 cm | Head | -0.1 to 0.0 s | 859 | -0.479 | 0.103 | -4.632 | 0.000 | 0.000 |
| 80 cm | Shoulder | -0.1 to 0.0 s | 859 | -0.326 | 0.082 | -3.976 | 0.000 | 0.001 |
| 80 cm | Pelvis | -0.1 to 0.0 s | 859 | -0.221 | 0.069 | -3.209 | 0.001 | 0.014 |
| Note; LMM results were obtained from the sensitivity analysis in which angular-difference outlier trials were retained, but angular-difference values exceeding the predefined outlier thresholds were capped at the corresponding threshold: 15.5° for the head, 18.7° for the shoulder, and 24.0° for the pelvis. Only time bins that remained significant after FDR correction are shown. | | | | | | | | |
